# Choice of Activity-Intensity Classification Thresholds Impacts upon Accelerometer-Assessed Physical Activity-Health Relationships in Children

**DOI:** 10.1371/journal.pone.0057101

**Published:** 2013-02-18

**Authors:** Daniel P. Bailey, Lynne M. Boddy, Louise A. Savory, Sarah J. Denton, Catherine J. Kerr

**Affiliations:** 1 Institute for Sport and Physical Activity Research (ISPAR), University of Bedfordshire, Bedford, Bedfordshire, United Kingdom; 2 The Research Institute for Sport and Exercise Sciences, Liverpool John Moores University, Liverpool, United Kingdom; University of Bath, United Kingdom

## Abstract

**Background:**

It is unknown whether using different published thresholds (PTs) for classifying physical activity (PA) impacts upon activity-health relationships. This study explored whether relationships between PA (sedentary [SED], light PA [LPA], moderate PA [MPA], moderate-to-vigorous PA, vigorous PA [VPA]) and health markers differed in children when classified using three different PTs.

**Methods:**

104 children (63 girls) aged 10–14 years wore an RT3 triaxial accelerometer for seven days and measures of adiposity and cardiometabolic risk markers were taken.

**Results:**

Significant associations (*p*< .05) in boys were found between LPA and body mass index *z*-score and waist circumference *z*-score for the Rowlands et al PT only (β =  .459 and.401, respectively) and body fat% (BF%) for the Chu et al PT only (β = .322) and in girls with BF% for the Rowlands et al PT only (β =  .303) and systolic BP and blood glucose for the Vanhelst et al PT only (β = −.298 and −.283, respectively). MPA was significantly (*p*<.05) associated with BF%, diastolic BP, and cardiorespiratory fitness (CRF) for the Chu et al PT only in girls (β = −.436, −.529, and .446, respectively). SED was significantly (*p*<.05) associated with triglycerides (β = .492) for the Rowlands et al PT only in boys and VPA with CRF (*p*<.05) for the Rowlands et al and Vanhelst et al PTs only in girls (β = .416 and .352, respectively).

**Conclusions:**

The choice of PT impacted upon activity-health relationships. A consensus on appropriate accelerometer thresholds for quantifying PA intensity and sedentary behaviour is needed in order to make accurate evidence-based recommendations for health promotion.

## Introduction

Childhood obesity has become a worldwide epidemic in recent decades [1] and associated cardiometabolic disorders, such as dyslipidaemia, hypertension, impaired glucose metabolism, and low cardiorespiratory fitness (CRF), also exist in this population [2], [3]. It is estimated that approximately 4.5% of US 12–17 year-olds have the metabolic syndrome [3] and this clustering of risk factors can persist into adulthood [4]. There has been considerable investment into research that explores the relationship between physical activity (PA), obesity and cardiometabolic health to inform intervention design and promote their efficacy [2], [5], [6].

To clearly understand the relationship PA has with health parameters, accurate and detailed PA data is essential. Accelerometry is the most common and one of the most effective methods for assessing free-living PA in children [7]. An accelerometer measures the acceleration of the body part it is attached to in one, two, or three planes for a specific predefined time period (epoch) and provides information on the frequency, duration and intensity of PA [7]. This data is used to explore the relationship between total PA or accumulated minutes of light PA (LPA), moderate PA (MPA), vigorous PA (VPA), moderate-to-vigorous PA (MVPA), or time spent sedentary (SED), and health markers [2], [8] and is also used to estimate the proportion of children meeting PA guidelines [9]. Chu et al [10] defined PA intensity according to MET values i.e. SED = < 2 METs, LPA≥2 to 3 METs, MPA≥3 to<6 METs and VPA≥6 METs, but this is not consistent across studies [11], [12].

Furthermore, despite widespread use of accelerometers, there is no standardised method for reduction of their data and the variations in which PA data is measured and expressed can affect outcome variables and conclusions within studies [13]. Accelerometers consist of piezoelectric transmitters that are stressed by accelerative forces, leading to the production of an electrical signal that is converted by processing units to produce an indication of movement. The outputs from accelerometers are dimensionless units commonly referred to as ‘counts’. Because these counts are arbitrary, energy expenditure calibration studies have been undertaken to give biological meaning to these data by providing cut points that correspond to various PA intensities [10], [11], [12]. This has resulted in a number of published count thresholds that can be used to estimate time spent sedentary and in different PA intensity categories. The RT3 (Stayhealthy, Inc.) is a triaxial accelerometer that integrates acceleration from three planes to yield a vector magnitude and this device has been used to assess free-living PA and associations with health markers in children [2], [14], [15], [16], [17]. However, the array of PA intensity thresholds available in the literature for use with the RT3 triaxial accelerometer [10], [11], [12] has led to inconsistency in the field, making it difficult to compare and interpret study findings.

In a recent review by Ekelund et al [9], the prevalence values for sufficiently active youth (i.e. accumulation of at least 60 min/day of MVPA) ranged between 1% and 100%. The authors outlined that differences in intensity thresholds were largely attributable to inconsistencies across studies. The influence of using different intensity thresholds on the associations observed between PA outcomes and health markers in children is much less clear due to a lack of empirical evidence. No study to date has investigated the impact that different PTs may have on the relationship between PA and health markers in children, which should be at the forefront of PA thresholds research given that the daily target for MVPA is based on observed associations with health outcomes [18].

The aim of this study was therefore to explore whether the relationships between PA subcomponents (SED, LPA, MPA, MVPA, and VPA) and health markers differ when PA intensity is classified using different PTs (Rowlands et al [11], Vanhelst et al [12] and Chu et al [10]) for the RT3 triaxial accelerometer. As shown in Table 1, the upper and lower limits for PA intensities differed considerably between the PTs investigated and it was thus hypothesised that activity-health relationships would differ dependent on PT employed.

**Table 1:** Activity-intensity thresholds for the RT3 accelerometer.

Variable	Rowlands [11]	Vanhelst [12]	Chu [10]
Sedentary (cpm)	<288	<41	<420
Light PA (cpm)	288–969	41–950	420–1859
Moderate PA (cpm)	970–2,332	951–3,410	1860–4109
Vigorous PA (cpm)	≥2,333	>3,410	≥4110

cpm, counts per min; PA, physical activity.

## Methods

### Ethics Statement

The Health And Physical activity Promotion in Youth (HAPPY) study received full ethical approval from the University of Bedfordshire ethics review board. Written informed consent was obtained from participants' parents and verbal assent from the participants before any testing procedures.

### Sample

The 104 participants (63 girls) included were part of the HAPPY study. This school-based study explored the effects of three interventions on PA levels and health outcomes in children and adolescents aged 10–14 years. Participants were recruited on a voluntary basis in 11 schools across Bedfordshire, UK, and baseline data were used for analyses in the present study. Participants were excluded if they had any contraindications to taking part in physical exercise. Parents were provided with their child's physiological results at the end of the study.

### Measurements

Age was recorded as a decimal value for each participant using date of birth on the date of testing. Stature was recorded to the nearest 0.5 cm using the portable Leicester Height Measure (Seca, Birmingham, UK). Body mass was recorded to the nearest 0.1 kg and body fat% (BF%) to the nearest 0.1% using the Tanita BC-418® Segmental Body Composition Analyser (Tanita Corp., Tokyo), which has been previously validated in boys and girls against whole-body dual X-ray absorptiometry and air-displacement plethysmography [19]. UK 1990 reference values were used to calculate *z*-scores for height, weight and body mass index (BMI) [20], [21] and McCarthy et al [22] reference values used to calculate *z*-scores for waist circumference (WC). Participants were required to fast from 9 pm the night before testing and measurements were taken between 8–10 am. Participants were instructed to bring a snack with them to eat for breakfast once testing had been completed.

Sitting blood pressure (BP) was measured (Omron M5-I automated oscillatory device, Omron Matsusaka Co. Ltd., Matsusaka, Japan) after the participant had rested for 5 min. Three BP readings were obtained, and the average for the lowest two readings recorded. Fasting blood samples were obtained using a finger prick method and were transferred into a cassette sample well and placed in the drawer of a Cholestech LDX analyser (Cholestech Corp., Hayward, CA., USA) to provide a valid measure of total cholesterol (TC), HDL, triglycerides, and blood glucose levels (*r* = 0.77–0.91 with core laboratory values) [23], [24].

To determine CRF, participants completed an age- and sex-specific all-out progressive cycle ergometer test to exhaustion using a previously validated protocol [25]. Briefly, workloads increased every 3 min until the participant was no longer able to continue. A maximal effort was deemed as a final heart rate≥185 beats per min (bpm) and subjective observation from the researcher that the child could not continue. Power output (watts) was calculated as being equal to W_1_+(W_2_ . t/180), where W_1_ is work rate at fully completed stage, W_2_ is the work rate increment at final incomplete stage, and t is time in seconds at final incomplete stage. VO_2max_ was calculated using previously described formula [25] and expressed relative to body mass (mL/kg/min).

RT3 triaxial accelerometers (Stayhealthy, Inc., Monrovia, CA., USA) were used to measure seven consecutive days of habitual PA using minute-by-minute sampling. The RT3 integrates acceleration and deceleration from three planes (vertical, anterioposterior and mediolateral vectors) to yield a vector magnitude, which is calculated as the square root of the sum of squared activity counts for each vector. To determine time in SED, LPA, MPA, MVPA, and VPA, intensity thresholds were based on Rowlands et al [11], Vanhelst et al [12] and Chu et al [10] calibration studies (see Table 1). Participants were only included for data analysis if they had worn the accelerometer for a minimum of three days [26] and acquired a minimum daily wear time of nine hours for weekdays [26] and eight hours for weekend days [27]. Sustained 10 min periods of zero counts were removed during the recoding process [28].

### Statistical analysis

All analyses were completed using SPSS version 18.0 (SPSS Inc., Chicago, IL., USA). Descriptive data are presented as mean (SD). Sex differences in descriptive variables were determined by one-way ANOVA. All subsequent analyses were conducted separately by sex. PA subcomponents were defined as SED^R^, LPA^R^, MPA^R^, MVPA^R^ and VPA^R^ when classified using the Rowlands et al [11] PT, SED^V^, LPA^V^, MPA^V^, MVPA^V^ and VPA^V^ when classified using the Vanhelst et al [12] PT and SED^C^, LPA^C^, MPA^C^, MVPA^C^ and VPA^C^ when classified using the Chu et al [10] PT. Differences in accumulated time for SED, LPA, MPA, MVPA and VPA according to each PT were examined using MANCOVA with age entered as a covariate. The proportion of boys and girls meeting current government recommendations of ≥60 min/day of MVPA [18] and previous suggestions of accumulating ≥90 min/day of MVPA to prevent insulin resistance [29] when using different PTs is also reported and binary logistic regression used to explore differences in MVPA recommendation compliance between PTs. The difference in blood glucose levels between boys and girls meeting the recommendation of 60 and 90 min/day of MVPA to those who did not was tested using ANCOVA with age entered as a covariate. Multiple linear regression analyses were used to explore associations between PA intensity (SED, LPA, MPA, MVPA and VPA) and health markers for each PT with age entered as an additional independent variable. SED^C^, LPA^V^, LPA^C^, MPA^R^, MPA^V^, MPA^C^, MVPA^V^, MVPA^R^, MVPA^C^, VPA^R^, VPA^V^, VPA^C^, BF%, TC:HDL ratio and triglycerides were non-normally distributed and were log10 transformed prior to analysis.

## Results

Table 2 shows the descriptive characteristics of the participants. One-way ANOVA revealed that *z*WC and BF% were significantly greater in girls versus boys, while CRF and total PA was significantly greater in boys. According to McCarthy et al [19] body fat reference curves for children, 10.6% of the sample were overweight and 4.8% were obese.

**Table 2:** Descriptive characteristics of participants.

	Boys (*N* = 41)	Girls (*N* = 63)
Age (yr)	11.78 (1.39)	11.76 (1.35)
*z*-height	0.33 (1.10)	0.44 (0.95)
*z*-weight	−0.03 (1.19)	0.22 (1.07)
*z*BMI	−0.41 (1.21)	−0.13 (1.37)
*z*WC	−0.19 (1.18)	0.42 (1.52)*
Body fat%	16.79 (5.74)	23.55 (5.60)**
Systolic BP (mm Hg)	106.1 (9.7)	105.0 (10.8)
Diastolic BP (mm Hg)	64.0 (7.3)	66.0 (6.7)
TC:HDL ratio	2.79 (0.70)	3.02 (1.08)
Triglycerides (mmol/L)	0.73 (0.33)	0.96 (0.72)
Blood glucose (mmol/L)	5.01 (0.39)	5.05 (0.51)
CRF (mL/kg/min)	45.70 (8.01)	38.50 (8.95)**
Total Physical activity (cpm)	486.93 (136.96)	416.92 (140.25)*

*z*BMI, BMI *z*-score; *z*WC, waist circumference *z*-score; BP, blood pressure; TC, total cholesterol; HDL, high-density lipoprotein cholesterol; CRF, cardiorespiratory fitness. *p<0.05, **p<0.001.

Girls spent significantly more time in SED than boys according to the Chu et al [10] PT. No other sex differences were revealed according to this PT. According to the Rowlands et al [11] PT, girls engaged in significantly more LPA and significantly less VPA than boys. According to the Vanhelst et al [12] PT, girls also engaged in significantly more LPA and significantly less VPA than boys as well as significantly less MVPA (see Table 3). 97.6% of boys and 93.7% of girls met the government recommendation of ≥60 min/day of MVPA when PA intensity was classified using the Rowlands et al [11] and Vanhelst et al [12] PTs, respectively, while only 31.7% of boys and 20.6% of girls met this recommendation when classified using the Chu et al [10] PT. Only 7.3% of boys and 6.3% of girls accumulated ≥90 min/day of MVPA according to the Chu et al [10] PT, while 80.5% of boys and 58.7% of girls achieved this level of PA according to Rowlands et al [11] and 78% of boys and 58.7% of girls according to the Vanhelst et al [12] PTs. The odds of accumulating ≥60 min/day of MVPA were significantly lower (*p*<.001) when using the Chu et al [10] PT compared with the Rowlands et al [11] and Vanhelst et al [12] PTs: odds ratio (OR) and 95% CI = .012 (.002, .055) in boys and .018 (.007, .045) in girls. The odds of accumulating≥90 min/day of MVPA were also significantly lower (*p*<.001) when using the Chu et al [10] PT compared with the Rowlands et al [11] and Vanhelst et al [12] PTs: OR and 95% CI = .021 (.006, .075) in boys and .048 (.016, .139) in girls. The odds of accumulating ≥60 and ≥90 min/day of MVPA did not differ (*p*>.05) between the Rowlands et al [11] and Vanhelst et al [12] PTs in boys or girls. Blood glucose levels were significantly lower in boys who achieved ≥90 min/day of MVPA compared to those who did not when using the Rowlands et al [11] PT (mean ± SD = 4.62±.38 and 5.14±.08 for those who did and did not achieve ≥90 min/day of MVPA, respectively; *F* = 5.95, *p* = .02) and Vanhelst et al [12] PT (4.62±.36 and 5.15±.07, respectively; *F* = 9.09, *p* = .005). No other significant differences were observed between boys and girls meeting or not meeting the ≥60 and 90 min/day of MVPA recommendations according to any PT.

**Table 3:** Time spent in each physical activity subcomponent (age entered as a covariate).

Variable	Rowlands [11]		Vanhelst [12]		Chu [10]	
	Boys	Girls	Boys	Girls	Boys	Girls
Sedentary (min)	430.7 (79.5)	458.2 (84.7)	188.7 (65.9)	201.5 (74.7)	470.9 (102.7)	522.9 (103.1)*
Light PA (min)	165.9 (30.5)	187.5 (45.3)*	396.4 (56.0)	437.8 (73.0)*	175.8 (41.6)	202.9 (114.0)
Moderate PA (min)	90.6 (30.3)	87.2 (30.4)	116.6 (51.2)	101.6 (40.1)	50.5 (23.1)	53.0 (123.4)
Vigorous PA (min)	28.6 (17.6)	19.7 (16.4)*	9.0 (9.2)	4.9 (6.0)*	3.7 (4.1)	17.9 (125.6)
MVPA (min)	119.2 (36.3)	105.5 (43.4)	125.6 (54.0)	106.5 (43.4)*	54.1 (23.1)	54.9 (123.5)

Data presented as mean (SD); PA, physical activity; MVPA, moderate-to-vigorous physical activity. *p<0.05 between sexes.

Total PA (mean cpm) was not significantly correlated with any health marker in either sex (*p*>.05). Table 4 shows associations between PA subcomponents and health markers in boys. None of the PA subcomponents were associated with any health marker according to the Vanhelst et al [12] PT. SED was significantly positively associated with triglyceride levels and LPA significantly positively associated with *z*BMI and *z*WC according the Rowlands et al [11] PT only. LPA was significantly negatively associated with CRF according to both the Rowlands et al [11] and Chu et al [10] PTs and significantly positively associated with BF% according to the Chu et al [10] PT only.

**Table 4:** Associations between physical activity subcomponents and cardiometabolic risk factors in boys according to different published thresholds for the RT3 triaxial accelerometer.

	Risk factor																	
	*z*BMI		*z*WC		Body fat%		CRF		Systolic BP		Diastolic BP		TC:HDL ratio		Triglycerides		Blood glucose	
	β	*p*	β	*p*	β	*p*	β	*p*	β	*p*	β	*p*	β	*p*	β	*p*	β	*p*
Rowlands [11]																		
SED	.107	.589	.133	.510	.122	.546	−.058	.764	.128	.527	.152	.447	.370	.076	**.492**	**.013**	−.207	.323
LPA	**.459**	**.018**	**.401**	**.041**	.350	.072	**−.491**	**.010**	.089	.639	.127	.500	**−**.025	.898	.278	.126	**−**.100	.609
MPA	**−**.187	.317	**−**.203	.289	**−**.080	.676	.167	.359	.185	.333	.258	.176	.073	.705	.250	.168	**−**.179	.365
VPA	.099	.568	.138	.436	.124	.486	**−**.136	.421	**−**.197	.267	**−**.204	.248	.102	.569	.226	.180	**−**.137	.454
Vanhelst [12]																		
SED	**−**.100	.623	**−**.063	.762	**−**.073	.717	.124	.541	.071	.735	**−**.061	.762	.284	.171	.228	.262	**−**.071	.737
LPA	.336	.064	.230	.215	.319	.077	**−**.279	.123	**−**.043	.813	.248	.165	.236	.196	.334	.065	**−**.126	.499
MPA	**−**.053	.793	**−**.107	.611	.019	.923	**−**.024	.906	.072	.732	.097	.632	.044	.829	.198	.330	**−**.077	.717
VPA	.125	.491	.168	.373	.122	.500	**−**.024	.894	**−**.181	.336	**−**.181	.319	.208	.262	.086	.633	**−**.144	.447
Chu [10]																		
SED	.109	.606	**−**.062	.717	.160	.431	.006	.975	**−**.061	.773	.002	.992	.100	.638	.228	.280	**−**.128	.543
LPA	.269	.113	.168	.332	**.322**	**.050**	**−.416**	**.010**	.037	.826	.110	.502	**−**.109	.516	.203	.226	.026	.876
MPA	.021	.930	**−**.034	.892	.040	.862	.091	.685	.022	.928	.137	.564	.058	.811	.121	.616	−.340	.162
VPA	−.112	.572	−.020	.923	−.126	.510	.127	.489	−.158	.429	−.336	.089	.228	.254	−.079	.688	−.019	.924

*z*BMI, BMI *z*-score; *z*WC, waist circumference *z*-score; CRF, cardiorespiratory fitness; BP, blood pressure; TC, total cholesterol; HDL, high-density lipoprotein cholesterol; SED, sedentary time; LPA, light physical activity; MPA, moderate physical activity; VPA, vigorous physical activity; significant associations highlighted in bold.

Table 5 shows associations between PA subcomponents and health markers in girls. LPA was significantly positively associated with BF% according to the Rowlands et al [11] PT only, while MPA was significantly negatively associated with BF% according to the Chu et al [10] PT only. LPA was significantly negatively associated with systolic BP and blood glucose according to the Vanhelst et al [12] PT only. MPA was significantly negatively associated with diastolic BP according to the Chu et al [10] PT only. LPA was significantly negatively associated with CRF according to the Rowlands et al [11] and Chu et al [10] PTs only. VPA was significantly positively associated with CRF according to the Rowlands et al [11] and Vanhelst et al [12] PTs, while MPA was significantly positively associated with CRF according to the Chu et al [10] PT only.

**Table 5:** Associations between physical activity subcomponents and cardiometabolic risk factors in girls according to different published thresholds for the RT3 triaxial accelerometer.

	Risk factor																	
	*z*BMI		*z*WC		Body fat%		CRF		Systolic BP		Diastolic BP		TC:HDL ratio		Triglycerides		Blood glucose	
	β	*p*	β	*p*	β	*p*	β	*p*	β	*p*	β	*p*	β	*p*	β	*p*	β	*p*
Rowlands [11]																		
SED	−.229	.161	−.313	.054	−.178	.267	.148	.327	−.236	.148	−.242	.146	−.044	.795	−.060	.718	−.086	.592
LPA	.212	.163	.249	.096	**.303**	**.044**	−**.324**	**.023**	−.172	.256	.159	.300	.182	.247	.017	.911	−.276	.069
MPA	−.112	.595	−.110	.593	−.301	.148	.149	.443	−.058	.780	−.255	.233	−.150	.492	.120	.574	.020	.924
VPA	−.076	.664	−.125	.469	−.027	.877	**.416**	**.012**	.093	.593	−.102	.567	−.029	.873	.133	.456	.201	.250
Vanhelst [12]																		
SED	−.262	.153	−.327	.075	−.233	.199	.184	.272	−.162	.360	−.179	.335	−.109	.554	−.032	.862	−.007	.969
LPA	−.014	.924	−.057	.693	.055	.703	−.159	.237	−**.298**	**.039**	−.084	.572	.105	.479	−.031	.831	−**.283**	**.046**
MPA	−.027	.881	−.004	.984	−.212	.238	.174	.296	.032	.855	−.156	.400	−.118	.520	.086	.635	.044	.800
VPA	−.093	.552	−.159	.309	−.039	.801	**.352**	**.016**	.037	.808	−.074	.641	−.105	.504	.203	.196	.217	.149
Chu [10]																		
SED	−.203	.167	−.274	.058	−.154	.284	−.078	.568	−.258	.077	−.239	.094	−.002	.990	−.229	.126	−.144	.320
LPA	.268	.150	.346	.059	.283	.123	−**.463**	**.010**	−.048	.794	.264	.145	.049	.797	.144	.446	−.212	.250
MPA	−.227	.285	−.282	.175	−**.436**	**.039**	**.446**	**.028**	−.029	.888	−**.529**	**.012**	−.021	.921	−.145	.503	−.002	.993
VPA	−.015	.941	−.097	.621	.097	.623	.186	.327	.041	.835	.094	.628	−.188	.363	.142	.488	.344	.089

*z*BMI, BMI *z*-score; *z*WC, waist circumference *z*-score; CRF, cardiorespiratory fitness; BP, blood pressure; TC, total cholesterol; HDL, high-density lipoprotein cholesterol; SED, sedentary time; LPA, light physical activity; MPA, moderate physical activity; VPA, vigorous physical activity; significant associations highlighted in bold.

## Discussion

This is the first study to explore the impact that using different published accelerometer thresholds has on activity-health relationships. It was revealed that associations between PA subcomponents and health markers differ markedly in children when PA intensity is classified using different published thresholds (PTs). This is important as there are a range of PTs available and indicates that studies using different PTs are unlikely to be comparable.

The relationship between PA intensity and CRF differed markedly between PTs. LPA was significantly negatively associated with CRF in boys and girls according to the Rowlands et al [11] and Chu et al [10] PTs but not the Vanhelst et al [12] PT. VPA was significantly positively associated with CRF in girls according to the Rowlands et al [11] and Vanhelst et al [12] PTs only and MPA significantly positively associated according to the Chu et al [10] PT only. The strength of the significant associations between LPA and CRF (moderate) and VPA and CRF (moderate) were similar across PTs. However, when significant associations were not consistent across PTs for PA subcomponents, the strength of associations tended to vary substantially. CRF was negatively associated with SED and positively associated with LPA, MPA and VPA in 9–10 and 15–16 year-old children in the European Youth Heart Study (EYHS) [8]. However, in 11–18 year-old youths, CRF was also positively associated with VPA, but unrelated to LPA and MPA [30] and it is possible that the use of different cpm thresholds may partly explain differences between studies. CRF has important cardioprotective effects and is consistently associated with lower risk of cardiovascular disease outcomes and mortality in adults [31] and is also favourably associated with cardiometabolic risk markers in youths [8]. Understanding the role of PA intensity for CRF is thus important and the current findings suggest that PA intensity-CRF relationships vary dependant on PT employed.

Other than CRF, VPA was not significantly associated with any other health marker according to any PT. In addition to CRF, MPA was significantly associated with diastolic BP in girls, but this was the case only when PA intensity was classified using the Chu et al [10] PT. The strength of association between PA subcomponents and diastolic BP also varied substantially across PTs. In the EYHS, there was also no significant association observed between MPA and VPA and triglycerides or levels of HDL [8]. Conflicting findings have been reported concerning other health markers, though, with significant negative associations observed between MPA and VPA and *z*BMI, WC, BF%, systolic BP, diastolic BP, and blood glucose [8], [15], [32], [33], [34]. Dissimilarities across studies may be due to differences in study population (i.e. different age ranges and sample size) but may also be explained by the use of different accelerometer devices (uniaxial MTI Actigraph vs. triaxial RT3). However, Hussey et al [15] also employed the Rowlands et al [11] PT used in the current study and reported significant associations between MPA, *z*BMI and WC in 7–10 year-old boys (no association in girls). Nonetheless, the observed associations may still have differed if an alternative PT had been employed.

In the case of time spent sedentary, this variable was only significantly (positively) associated with one health marker; triglycerides. However, this was the case only when classified using the Rowlands et al [11] PT. Conflicting findings have been reported previously with significant positive associations between SED and other cardiometabolic risk markers (systolic BP, diastolic BP, and blood glucose) [8]. However, relationships between SED and adiposity markers are less consistent [15], [35]. Hussey et al [15] employed the Rowlands et al [11] PT for use in their study and, contrary to the current findings, found a significant positive association between SED and WC in boys, which might suggest factors other than differences in PTs employed explain differences across studies. However, the current study explored associations between WC *z*-score and PA subcomponents, whereas Hussey et al [15] investigated raw WC data only.

In boys, LPA was significantly positively associated with BF% only when classified using the Chu et al [10] PT. Taken alone, this observation might suggest that LPA is detrimental to health if, as previously suggested [36], LPA replaces engagement in PA of higher intensities. However, there was no significant negative association between MPA and VPA with BF% according to any of the PTs. In the EYHS there was no association between LPA and WC in 1,709 children [8], although, unlike the current study, WC was not expressed relative to age and sex. Furthermore, LPA^V^ was significantly negatively associated with systolic BP and blood glucose in girls in the current study, while LPA was positively associated with CRF in the EYHS [8]. These data suggest that evidence concerning the relationship that LPA has to health in children is conflicting, requires further research, and may be affected by the PTs employed to classify PA intensity.

As stated, LPA was significantly negatively associated with systolic BP and blood glucose in girls, but only according to the Vanhelst et al [12] PT. Significant negative associations between LPA and systolic BP were also reported in the EYHS, although no association was observed with blood glucose [8]. The choice of published intensity thresholds thus appears to impact upon the associations between LPA and health markers in children. This is an important observation as the use of different PTs may hinder the development of a robust evidence-base from which to develop and evaluate prevention and treatment strategies. It is unclear why sex differences were observed between PTs e.g. there was a significant association between SED and triglycerides in boys according to the Rowlands et al [11] PT but no significant association between SED and triglycerides in girls according to any PT. Previous studies have also reported differences between sexes in the association between PA subcomponents and health markers [15]. This is the first study to demonstrate such differences according to sex and PT and highlights further the need for a consensus PT to better understand the association of PA to health in both boys and girls.

In general, the strength of association between PA subcomponents and health markers differed considerably across PTs. This is highlighted by the observation that no PA subcomponent was significantly associated with a health marker (other than CRF) for more than one PT. However, the strength and direction of associations tended to be more similar for MPA and VPA according to the Rowlands et al [11] and Vanhelst et al [12] PTs in comparison to the Chu et al [10] PT. This is likely due to the cpm for MPA and VPA being more similar for these two PTs in comparison to the Chu et al [10] PT, although a much larger sample size would be needed to detect significant differences between the strength of associations tested.

In addition to exploring relationships between PA intensity and health, another important use of accelerometers is to determine the amount of time youths spend being physically active [9]. Current government guidelines state that children and young people should engage in at least 60 min of MVPA every day [18]. It is established that PA intensity thresholds impact upon the prevalence of sufficiently active youth, as evidenced in a review by Ekelund et al [9] who demonstrated that the proportion of youth meeting the government recommendation ranges between 1% to 100%, dependent on intensity threshold used, when determined using accelerometry. The proportion of boys and girls meeting government recommendations in the current study was 97.6% and 93.7%, respectively, when PA intensity was classified using the Rowlands et al [11] and Vanhelst et al [12] PTs. This observation can be attributed to the similar lower limit cpm used to define MVPA between the two PTs: 970 and 951 cpm according to Rowlands et al [11] and Vanhelst et al [12], respectively. In light of previous research [37], though, it seems unlikely that such a high proportion of children would be sufficiently active or engage in approximately two hours of MVPA per day (see Table 3) and these lower thresholds for MVPA may thus be inaccurate. Indeed, according to the Chu et al [10] PT, the proportion of boys and girls achieving ≥60 min/day of MVPA was substantially lower (31.7% and 20.6%, respectively), which reflects the lower cpm threshold used, which may thus be more appropriate when measuring PA levels in children. The proportion of boys and girls achieving ≥ 90 min/day of MVPA, which may be the amount of PA needed to prevent insulin resistance in this population [29], was also substantially lower according to the Chu et al [10] PT compared to the Rowlands et al [11] and Vanhelst et al [12] PTs.

As the proportion of children meeting government recommendations for engagement in PA(≥60 min/day of MVPA) was unexpectedly high and possibly inaccurate according to the Rowlands et al [11] and Vanhelst et al [12] PTs, this may suggest that these two PTs are also unsuitable for use when exploring associations between PA and health. The Chu et al [10] PT may thus be preferable and offer a more accurate representation of the role of PA for health in this population given that the proportion of children meeting government recommendations for PA according to this PT appeared more realistic. However, further research of a longitudinal nature is needed to further explore this hypothesis.

The activities that children performed, the data reduction methods and the sample population i.e. age and country of residence, differed between calibration studies for the PTs investigated in the current research. These may all be explanatory factors for the differences in generated thresholds between calibration studies. There were also differences in the definition of PA intensities. For example, SED corresponded to <2 METs in the Chu et al [10] calibration study and to <1.5 METs in the Rowlands et al [11] study. MPA corresponded to ≥3 and <6 METs for both the Chu et al [10] and Rowlands et al [11] PTs and VPA ≥ 6 METs. Unfortunately, the Vanhelst et al [12] calibration study did not report the MET values that PA intensities corresponded to and ideally PTs would be compared for the same intensity. The range of existing PTs for the RT3 and other accelerometer models that differ in their thresholds to define PA intensity has been a shortcoming in PA prevalence research and policy making for children, thus emphasising the need for a consensus on appropriate accelerometer thresholds to quantify PA and appropriately focus future strategy efforts.

PA is highly variable in youth and the minimum criteria of three days accelerometry measurement may not fully capture a representative snapshot of this complex behaviour [38], while the minimum daily wear time of 9 h for a week day and 8 h for a weekend day may not fully capture time spent in SED and LPA. One limitation of this study may thus be that the magnitude of association between PA subcomponents and health markers is underestimated. This study is also limited by a relatively small sample size (*N* = 104) that may have weakened the strength of associations observed. The study also included only schools in Bedfordshire, UK and the proportion of physically active youth and the associations observed between PA intensities and health markers may thus not be representative of the UK population.

In conclusion, the use of different published thresholds to classify PA intensity impacts upon activity-health relationships in boys and girls. This suggests that studies using different PTs to investigate the associations between PA and health and the prevalence of sufficiently active youth may not be clearly comparable. In order to make accurate evidence-based recommendations and implement effective strategies to promote health in children, a consensus on appropriate accelerometer thresholds for quantifying PA intensity is needed.
